# Transcriptome sequencing of neurologic diseases associated genes in HHV-6A infected human astrocyte

**DOI:** 10.18632/oncotarget.10127

**Published:** 2016-06-17

**Authors:** Qing Shao, Zhe Lin, Xiaohui Wu, Junwei Tang, Shuai Lu, Dongju Feng, Ci Cheng, Lanqun Qing, Kun Yao, Yun Chen

**Affiliations:** ^1^ Department of Immunology, Nanjing Medical University, Nanjing, Jiangsu Province, People's Republic of China; ^2^ Department of Ophthalmology, The First Affiliated Hospital of Nanjing Medical University, Nanjing, Jiangsu Province, People's Republic of China; ^3^ Genetic Data Analysis Group, Shanghai Biotechnology Corporation, Shanghai, People's Republic of China; ^4^ Liver Transplantation Center of The First Affiliated Hospital and Collaborative Innovation Center For Cancer Personalized Medicine, Nanjing Medical University, Nanjing, Jiangsu Province, People's Republic of China

**Keywords:** HHV-6, astrocyte, transcriptome sequencing study, neurologic diseases

## Abstract

Human Herpesvirus 6 (HHV-6) has been involved in the development of several central nervous system (CNS) diseases, such as Alzheimer's disease, multiple sclerosis and glioma. In order to identify the pathogenic mechanism of HHV-6A infection, we carried out mRNA-seq study of human astrocyte HA1800 cell with HHV-6A GS infection. Using mRNA-seq analysis of HA1800-control cells with HA1800-HHV-6A GS cells, we identified 249 differentially expressed genes. After investigating these candidate genes, we found seven genes associated with two or more CNS diseases: CTSS, PTX3, CHI3L1, Mx1, CXCL16, BIRC3, and BST2. This is the first transcriptome sequencing study which showed the significant association of these genes between HHV-6A infection and neurologic diseases. We believe that our findings can provide a new perspective to understand the pathogenic mechanism of HHV-6A infection and neurologic diseases.

## INTRODUCTION

Human Herpesvirus 6 (HHV-6) exists as two related herpes viruses, HHV-6A and HHV-6B, that infect almost all human beings, especially the children [1, 2]. HHV-6 has a life-long latency and can become reactivated infection later [3]. HHV-6 reactivation has been linked with many clinical appearances throughout the body, including the lungs, kidney, heart, brain, and gastrointestinal tract [4, 5]. HHV-6 can infect various CNS cells in vitro [6–10]. HHV-6 has been involved in the progress of various range of neurologic disorders, including encephalitis, seizures, chronic fatigue syndrome, mesial temporal lobe epilepsy (MTLE), Alzheimer's disease, and multiple sclerosis [11, 12]. The diverse pathology may due to the viral sequence variations and differences in antigenic specificity between the HHV-6A and HHV-6B [11]. More research is needed to understand the important disease associations that have been suggested.

Recently, mRNA-seq has been increasingly used to explore the genetic and environmental factors of virus infection and diseases occurrence. Here, we undertook a genome-wide survey to map cellular genes of human astrocyte HA1800 that are infected by HHV-6A GS. In this study, we report the identification and comparative analysis of the differentially expressed genes that occurred during the virus infection phenotype conversion process. Therefore, this work is the first attempt at evaluating, genome-wide, the genotype-to-transcriptome-to-clinical phenotype associations in HHV-6A GS infection diseases.

## RESULTS

### Analysis of differentially expressed genes (DEGs)

**Figure 1:**
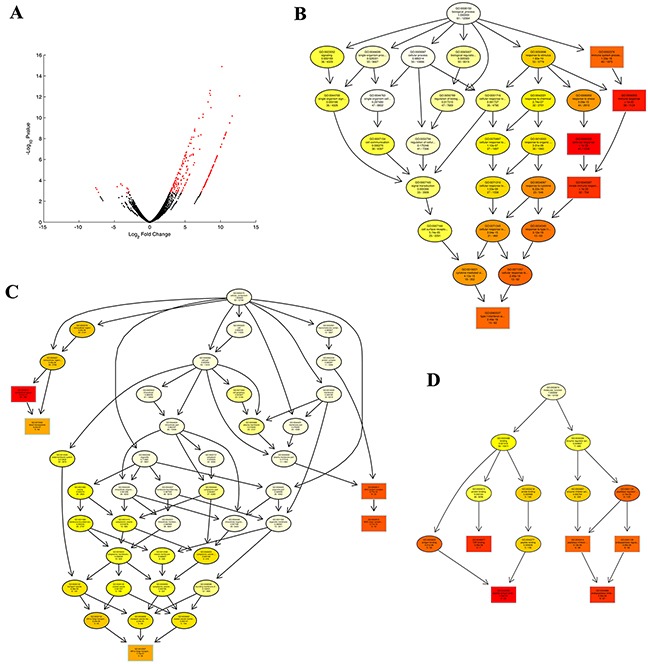
Differentially expressed genes (DEGs) enriched and identified by GO analyses **A.** The expression of cellular genes in two enriched populations of HA1800-control and HA1800-HHV6GS cells for 24 h were assessed using mRNA-seq. The distribution of genes with a change in expression of false discovery rate (FDR) < 0.1 is shown in red on the MA plot (log total counts versus log fold-change). **B.** The biological processes of the DEGs were identified by GO analyses. **C.** The cellular components of the DEGs were identified by GO analyses. **D.** The molecular functions of the DEGs were identified by GO analyses.

The potential DEGs (16430 genes, and 249 genes of FDR < 0.1) between different groups are displayed in Supplementary Table S1. The potential DEGs with FDR < 0.1 (HA1800-control expression > −1 and HA1800-HHV6-GS expression > 0) between libraries are presented in Figure 1A and Supplementary Table S2. Totals of 66 significant DEGs (only 8 genes are downregulated) were identified during the HHV-6A GS virus infection human astrocyte HA1800.

### Gene ontology analysis of potential DEGs

In order to obtain further understanding of the biological functions of the DEGs, Gene Ontology (GO) analyses were performed. We chose significant GO categories listed in Supplementary Table S3, S4, and S5. The biological processes, cellular component, and molecular function are presented in Figure 1B, 1C, and 1D, respectively. And, potential DEGs were enriched for GO categories of defense response (GO:0006952), immune response (GO:0006955), innate immune response (GO:0045087), immune system process (GO:0002376), type I interferon signaling pathway (GO:0060337) in biological processes; extracellular space (GO:0005615), MHC class I protein complex (GO:0042612), blood microparticle (GO:0072562) in cellular component; peptide antigen binding (GO:0042605), endopeptidase inhibitor activity (GO:0004866) in molecular function.

### Signaling pathway analysis of potential DEGs

**Figure 2:**
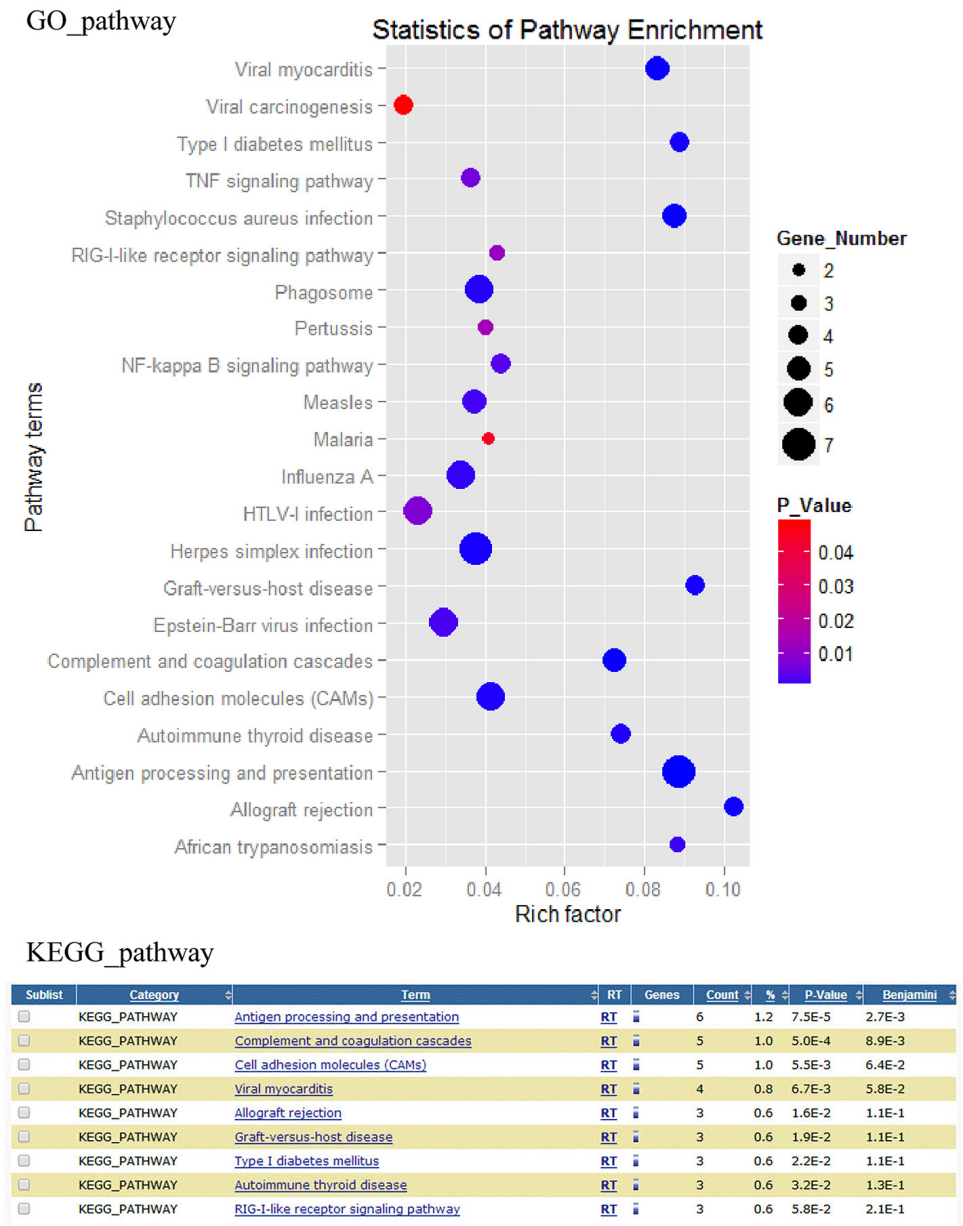
Differentially expressed genes associated pathways analysis Differentially expressed genes associated pathways were analyzed by GO and KEGG pathway tools.

All signaling pathways of DEGs were showed in Figure 2, and the important signaling pathway classes were listed in Supplementary Table S5. According to the results of the KEGG and GO pathway analysis, we focus on the DEGs involved in viral carcinogenesis, viral myocarditis, HTLV-1 infection, Epstein-Barr virus infection, influenza A, herpes simplex infection, TNF signaling pathway, RIG-I-like receptor signaling pathway, NF-kappa B signaling pathway, graft-versus-host disease, complement and coagulation cascades, autoimmune thyroid disease, antigen processing and presentation, allograft rejection, measles and phagosome signaling pathways.

### CNS diseases association analysis of potential DEGs

**Figure 3:**
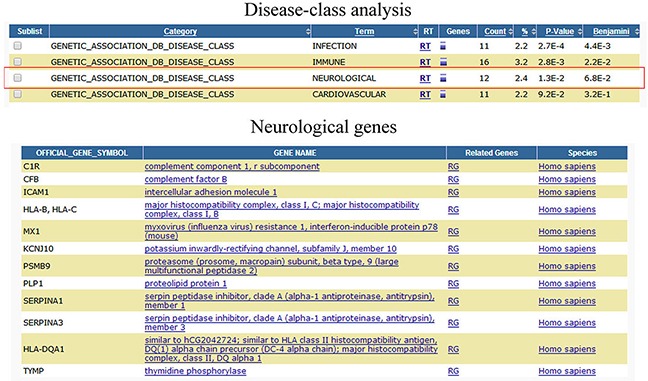
Differentially expressed genes associated CNS diseases analysis Differentially expressed genes associated CNS diseases were analyzed by DAVID functional annotation chart tool.

**Figure 4:**
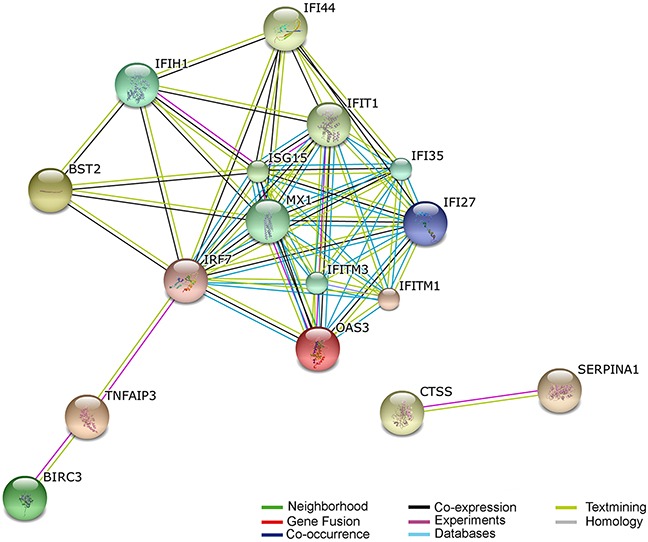
Predicted interaction networks of genes differentially expressed during HHV-6A infection Differentially expressed genes are depicted: links have been predicted using STRING (http://string.embl.de/). Predicted interactions are depicted according to the type of available evidence. The interactions (see color labels) include direct (physical) and indirect (functional) associations; they are derived from four sources: genomic context, high-throughput experiments, conserved coexpression, and previous knowledge from literature.

All disease classes of DEGs were analyzed by functional annotation chart tool (https://david.ncifcrf.gov/home.jsp) [13, 14] in Figure 3 (and Supplementary Figure S1). And the differentially expressed genes involved in infection, immune, neurological, and cardiovascular disease classes. We then further analyzed which of the potential DEGs associated with antivirus, Alzheimer's Disease, glioma, and multiple sclerosis following HHV-6A GS virus infection human astrocyte HA1800. Of these genes, 12 were associated with antivirus function; 7 were associated with Alzheimer's Disease; 11 were associated with glioma; 9 were associated with multiple sclerosis (Table 1). And more importantly, CTSS, PTX3, CHI3L1, Mx1, CXCL16, BIRC3, and BST2 genes exhibited significant correlation with more than two CNS diseases. Subsequently, these genes were further recognized by real-time PCR assay in cells at 24 hours and 72 hours (Supplementary Figure S2A and S2B). And the expression of CTSS, Mx1, and BIRC3 genes were further validated by western blot assay at 72hours (Supplementary Figure S2C). The main genes revealing a positive association with viral infection by using the STRING database (i.e., increasing gene expression with viral infection) was shown in Figure 4 (and Supplementary Figure S3). The STRING database (http://string-db.org) designs to supply an important tool for studying protein–protein interactions, including direct and indirect correlations.

**Table 1:** the differentially expressed genes associated with antivirus, alzheimer's disease, glioma, and multiple sclerosis

CNS diseases	Representative DEGs in HA1800-HHV6 GS/control
**Antivirus**	OAS3, LGALS9, IFI35, BIRC3, IFI44, IFITM3, IRF7, IFIT1, IFITM1, IL32, IFI27, ISG15
**Alzheimer's disease**	CTSS, SERPINA1, NPTX1, PTX3, CHI3L1, SERPINA3, Mx1
**Glioma**	CTSS, IRF7, CXCL16, IFITM3, PTX3, CHI3L1, TNFAIP3, BIRC3, BST2, IFIT1, IFITM1
**Multiple sclerosis**	CHI3L1, IFIH1, KCNJ10, SERPINA1, CXCL16, IFITM3, PTX3, BST2, Mx1

## DISCUSSION

Genome-wide gene expression profiling is an impartial method to ascertain the pathogenic mechanism underlying virus infection. Our study shows many differentially expressed genes, which related to pathogenic mechanism, are upregulated in HA1800 cells with HHV-6A infection as compared with uninfected controls. The mRNA-Seq technology has no hybridization bias and almost no batch effects on gene expression [15].

Antiviral genes are activated in HA1800 cells with HHV-6A infection. In addition, new effects of anti-viral can be disclosed via comparative analysis of genome-wide transcriptional profiles. OAS3 p100 employs host antiviral effect against Chikungunya virus [16], dengue virus infection [17], and HCV [18]. Galectin-9 (LGALS9) is rapidly released during acute HIV-1 infection [19]. IFN-inducible protein 35 (IFI35) plays an important role in the type I interferon response induced by foot-and-mouth disease virus protein 2C [20] and in the maintenance of foamy virus latency [21] and vesicular stomatitis virus replication [22]. Baculoviral IAP repeat containing 3 (BIRC3) inhibited hepatitis B virus replication [23]. BIRC3 upregulated by E6 oncoprotein confers resistance to cisplatin in human papillomavirus 16/18-infected lung cancer [24]. Interferon-induced protein 44 (IFI44) inhibits HIV-1 LTR promoter activity [25]. IFITM3 restricts influenza A virus entry [26] and has susceptibility to respiratory viral infection [27]. IFITM3 also restricts reovirus cell entry [28] and morbidity and mortality associated with influenza [29]. Lysine residues of interferon regulatory factor (IRF7) affect the replication of KSHV [30]. IFIT1 (ISG56) recognizes 5′-triphosphate RNA [31]. Interferon induced transmembrane protein 1 (IFITM1) restrict membrane fusion [32]. Interferon, alpha-inducible protein 27 (IFI27, ISG12a) is IFN-induced protein that impact cellular apoptosis [33]. High basal ISG12a may inhibit NDV replication and oncolysis [34]. ISG15 inhibits the replication of influenza A virus [35] and the Japanese encephalitis virus [36] and controls the proinflammatory response against viral infection [37]. The consecutive study of these genes in astrocytes can provide new clue for the elucidating of viral antagonism in HHV-6A infection.

HHV-6 showed 23% positivity in peripheral blood leukocytes samples from Alzheimer's disease (AD) and 4% from controls. HHV-6 may be environmental risk factors for cognitive deterioration and progression to AD in elderly persons [38–41]. We identified a number of AD related genes in astrocyte HA1800 cells with HHV-6A GS infection. Cathepsins S (CTSS) was evaluated as therapeutic target to develop disease modifying drugs to treat AD [42]. Serpin peptidase inhibitor, clade A, member 1 (SERPINA1, ATT) associates with AD-related phenotypes [43] and is recognized as biomarker [44] and potential indicator [45] for AD. Neuronal pentraxin 1 (NPTX1) is overexpressed in dystrophic neurites in AD [46]. Pentraxin-3 (PTX3) is an inflammatory marker [47] and its plasma levels are increased in patients with Parkinson's disease [48]. PTX3 is also a putative AD biomarker and pharmacological therapeutic target [49]. Cerebrospinal fluid level of chitinase 3-like 1 (CHI3L1, YKL-40) protein is elevated in AD [50–54] and could track the inflammatory processes in AD [55]. YKL-40 has potential prognostic utility as a biomarker for preclinical AD [56]. SERPINA3 (ACT) polymorphism may affect age-at-onset and disease duration of AD [57]. The appearance of MX dynamin-like GTPase 1 (Mx1, MxA) protein in reactive microglia contributes to AD pathology [58]. Therefore, it is conceivable that these genes, combined with previously known mechanisms, may contribute to discovering the correlation between HHV-6A infection and the progression of AD.

HHV-6 DNA was detected in 86% of Nodular Sclerosis Hodgkin lymphoma (NSHL) cases. It suggests that HHV-6 may play an important role in NSHL pathogenesis [59] High percentages of HHV-6 DNA and protein were found in glioma tissue. Additionally, a strain of HHV-6A was isolated from the fluid specimens from glioma cysts. Our previous studies strongly show that HHV-6 infection is involved in the pathogenesis of glioma [60]. Activation of HHV-6 may lead to decrease of lymphocytes total count and develop immunosuppression in patients with gastrointestinal cancer [61]. Cathepsin S (CTSS) expression is linked with tumor progression and poor outcome in glioblastomas [62]. Interferon regulatory factor 7 (IRF7) can enhance glioma cell invasion, chemoresistance, and radioresistance [63]. CXCL16 is highly expressed by glial tumor and stroma cells in glioma [64]. Interferon induced transmembrane protein 3 (IFITM3) plays an important role in glioma cell growth and migration [65]. Pentraxin 3 (PTX3) was significantly associated with the presence of a high-grade glioma tumor [66]. Elevated expression of chitinase 3-like 1 (CHI3L1, YKL-40) in glioma was correlated with decreases in disease survival [67, 68]. YKL-40 serum values were markedly higher in glioma patients than in healthy subjects [69], and as potential serum biomarker for patients with high-grade glioma [70]. TNF alpha induced protein 3 (TNFAIP3, A20) is a tumor enhancer in glioma [71] and inhibits apoptosis in glioblastoma [72]. A20 may serve as a future therapeutic target [73]. BIRC3 (c-IAP2) facilitates cancer cell survival [74]. Increased c-IAP2 expression was found to enhance IκB-α phosphorylation in GBM cells [75, 76]. BST2 expression is upregulated in high grade human astrocytoma [77–79]. And BST-2 expression was increased once oncogenesis is initiated [80]. IFIT1 (ISG56) expression increases in U373MG human astrocytoma cells. [81]. IFITM1 expression significantly inhibited proliferation, migration, and invasion of glioma [82–84].

HHV-6 has been suggested in several autoimmune diseases, including multiple sclerosis (MS). HHV-6 (especially HHV-6A) could participate in neuroinflammation in MS via promoting inflammatory processes through CD46 binding [85]. Viral load and IgGs reacting with HHV-6 U94/REP protein were significantly higher in MS patients. [86]. In addition, anti-HHV-6 IgG was found in CSF of MS patients [87]. HHV-6 may have a role in long-term infection with demyelination in progressive neurological diseases [88]. Cerebrospinal fluid level of chitinase 3-like 1 (CHI3L1, YL-40) is induced in astrocytes in a variety of neurological diseases [89]. YKL-40 has been proposed as a biomarker of multiple sclerosis [90]. CSF level of YKL-40 is increased in MS [91] and is a prognostic marker in MS [92]. Enhanced skin expression of IFIH1 (MDA5) in dermatomyositis and related autoimmune diseases [93]. KCNJ10 (KIR4.1) is expressed in oligodendrocytes and astrocytes in the adult human brain. [94]. Significant expression differences of SERPINA1 (AAT) were identified as potential disease signatures for MS patients [95] and elevates in the cerebrospinal fluid of patients with MS [96]. CXCL16 could be a novel biomarker and potential predictor of disease activity in MS [97]. IFITM3 leads to neuropathological impairments and brain dysfunction in astrocytes [98]. Pentraxin 3 (PTX3) is a novel biomarker of inflammatory in MS [99]. Bone marrow stromal cell antigen 2(BST2) associated statistically with the risk of getting MS. [100]. The appearance of MX dynamin-like GTPase 1 (Mx1, MxA)mRNA is related to clinical exacerbations of MS. [101].

HHV-6 is a global virus in the adult population and correlated with several neurologic diseases, including Alzheimer's disease, glioma, and multiple sclerosis in the CNS. In conclusion, based upon the results of our comprehensive analysis of HHV-6A infected HA1800 cells, we revealed several genes correlated with neurologic disorders, especially CTSS, PTX3, CHI3L1, Mx1, CXCL16, BIRC3, and BST2 genes. Our studies highlight the human astrocyte HA1800 infected with HHV-6A GS virus and may enhance the understanding of the HHV-6A pathogenicity. The next challenge is to conduct further studies in revealing the role of these genes under HHV-6A infection.

## MATERIALS AND METHODS

### Cell culture

Cord blood mononuclear cells (CBMCs) were purified from the cord blood samples obtained from the Affiliated Women and Children Hospital of Nanjing Medical University. These studies were approved by the local ethics committee and institutional review board. All samples were obtained with consent from patients and volunteers. HSB-2 cell line (ATCC, USA) was cultured in 1640 medium (Gibco, USA) containing 10% fetal calf serum (FCS, Gibco, USA). Primary human fetal astrocyte HA1800 were purchased from the Sciencell company (Carlsbad, CA, USA) and cultured in DEME/F12 medium (Gibco, USA) supplemented with 10% FCS.

### Infection of astrocyte by the isolated HHV-6A GS

HA1800 (2 × 10^5^/well) were cultured in 6-well plates and then infected with the HHV-6A GS at a multiplicity of infection of 100. The culture HA1800 cells were collected for mRNA-seq at 24 hours after infection. The detail experiments were performed as previous described [60, 102–106].

### RNA sequencing and data analysis

The six samples (three HA1800-CTL and three HA1800-HHV6AGS samples) were shipped to the GENEWIZ Company (www.genewiz.com) for library construction and mRNA-Seq. Sequencing library construction included these steps: RNA quality checking (Agilent 2100, Agilent Eukaryote Total RNA Nano Kit), library construction (Illumina TruSeq RNA Sample Pre Kit), library purification (Beckman AMPure XP beads), insert fragments test (Agilent 2100, Agilent High Sensitivity DNA Kit), quantitative analysis of library (ABI 7500 real time PCR instrument; KAPA SYBR green fast universal 2×9 qPCR master mix, GRN), and cBOT automatic cluster (TruSeq PE Cluster Kit v3-cBotHS). High-throughtput sequencing was performed with Illumina HiSeq 2000. mRNA-Seq data analysis consisted of the following steps: data quality checking using the Fastqc software (http://www.bioinformatics.babraham.ac.uk/projects/fastqc/) and removing excess adaptors to get high-quality and clean reads; mapping the high-quality reads to the poplar tree reference genome (http://www.ncbi.nlm.nih.gov/genome/51?genome_assembly_id=273342), using the TopHat software (version 2.0.9) (Trapnell et al.2012); transcript assembling and expression quantificationusing Cufflinks (version 2). Gene expression was expressed as fragments per kilo-base transcript per million mapped reads (FPKM).

### Real-time PCR

RNAs were extracted from cells using TRIzol (Invitrogen, California, USA) kit according to the manufacturer's instructions. Subsequently, total RNA was reverse transcribed using SuperScript III reverse transcriptase (Invitrogen, California, USA). Real-time PCRs were then performed in ABI PRISM7500 system (Applied Biosystems, California, USA), according to the manufacturer's instructions. The expression level of each gene was normalized by GAPDH and reported as relative levels. The primers for real-time PCR were shown in Supplementary Table S7.

### Western blot

Whole cells were washed in PBS and lysed in RIPA lysis buffer supplemented with protease inhibitor cocktail (Roche, Mannheim, Germany). Total protein was quantified using a BCA Protein Assay Kit (Beyotime, Jiangsu, China), and equal amounts of whole cell lysates were resolved by SDS-polyacrylamide gel electrophoresis (PAGE) and transferred to a polyvinylidene difluoride (PVDF) membrane (Millipore, Eschborn, Germany). The blots were blocked with BSA (5% w/v in PBS) for 1 h at room temperature. The following primary antibodies were applied according to the manufacturer's instructions. Anti-Cathepsin S (CTSS, ab135651), Anti-MX1 (Mx1, ab95926), Anti-cIAP2 antibody (BIRC3, ab32059), Anti-GAPDH antibody (ab8245) were purchased from Abcam (Cambridge, MA, USA). The appropriate secondary antibodies were used at 1:2,000-1:5,000 (v/v) dilutions in PBS + 0.1% Tween 20 for 1 h at room temperature, and the signals were revealed using ECL kit (Thermo Scientific Pierce, Rockford, USA).

### Pathway and network analyses

The Search Tool for the Retrieval of Interacting Genes/Proteins (STRING) (http://string.embl.de/) was used to identify known and predicted interactions (derived from four sources: genomic context, high-throughput experiments, co-expression, and previous knowledge). DAVID Bioinformatic resources (http://david.abcc.ncifcrf.gov/) using the annotation sources GOTERM-BP (biological process), and GOTERM-MF (molecular function) identified functional categories.

## SUPPLEMENTARY FIGURES AND TABLES














