# Supplementary material for: Transcriptome sequencing of neurologic diseases associated genes in HHV-6A infected human astrocyte
Source: Oncotarget. 2016 Jun 17;7(30):48070–80. doi: 10.18632/oncotarget.10127 (PMC5217001; doi:10.18632/oncotarget.10127)
Supplement: Supplementary file 1 [file oncotarget-07-48070-s001.pdf]

Transcriptome sequencing of neurologic diseases associated genes in HHV-6A infected human astrocyte

SUPPLEMENTARY FIGURES AND TABLES

Disease analysis

Sublist	Category	Term	RT	Genes	Count	%	P-Value	Benjamini
	GENETIC_ASSOCIATION_DB_DISEASE	diabetes, type 1	RT		9	1.8	3.5E-4	1.0E-1
	GENETIC_ASSOCIATION_DB_DISEASE	hepatitis B	RT		5	1.0	6.5E-4	9.6E-2
	GENETIC_ASSOCIATION_DB_DISEASE	bone marrow transplantation	RT		4	0.8	1.2E-3	1.2E-1
	GENETIC_ASSOCIATION_DB_DISEASE	hepatitis C, chronic	RT		4	0.8	6.2E-3	3.8E-1
	GENETIC_ASSOCIATION_DB_DISEASE	Graves disease	RT		3	0.6	9.8E-3	4.6E-1
	GENETIC_ASSOCIATION_DB_DISEASE	cholangitis, sclerosing	RT		3	0.6	1.5E-2	5.5E-1
	GENETIC_ASSOCIATION_DB_DISEASE	multiple sclerosis	RT		6	1.2	1.7E-2	5.4E-1
	GENETIC_ASSOCIATION_DB_DISEASE	tuberculosis	RT		4	0.8	1.8E-2	5.1E-1
	GENETIC_ASSOCIATION_DB_DISEASE	psoriasis	RT		4	0.8	2.5E-2	5.9E-1
	GENETIC_ASSOCIATION_DB_DISEASE	lung cancer; Lambert-Eaton myasthenic syndrome	RT		2	0.4	2.8E-2	6.0E-1
	GENETIC_ASSOCIATION_DB_DISEASE	ankylosing spondylitis	RT		3	0.6	3.3E-2	6.1E-1
	GENETIC_ASSOCIATION_DB_DISEASE	multiple sclerosis; optic neuritis	RT		2	0.4	4.7E-2	7.2E-1
	GENETIC_ASSOCIATION_DB_DISEASE	nasopharyngeal cancer	RT		3	0.6	4.8E-2	6.9E-1
	GENETIC_ASSOCIATION_DB_DISEASE	rheumatoid arthritis	RT		5	1.0	5.1E-2	6.9E-1
	GENETIC_ASSOCIATION_DB_DISEASE	Plasmodium falciparum infection	RT		2	0.4	5.6E-2	7.0E-1
	GENETIC_ASSOCIATION_DB_DISEASE	IgA, IgD, IgG, IgM	RT		2	0.4	5.6E-2	7.0E-1
	GENETIC_ASSOCIATION_DB_DISEASE	HIV; cytomegalovirus retinitis	RT		2	0.4	5.6E-2	7.0E-1
	GENETIC_ASSOCIATION_DB_DISEASE	cytokine resposne	RT		2	0.4	5.6E-2	7.0E-1
	GENETIC_ASSOCIATION_DB_DISEASE	diabetes, latent autoimmune	RT		2	0.4	5.6E-2	7.0E-1
	GENETIC_ASSOCIATION_DB_DISEASE	graft-versus-host disease	RT		3	0.6	7.1E-2	7.6E-1
	GENETIC_ASSOCIATION_DB_DISEASE	measles	RT		2	0.4	8.3E-2	8.0E-1
	GENETIC_ASSOCIATION_DB_DISEASE	infection, post allograft	RT		2	0.4	8.3E-2	8.0E-1
	GENETIC_ASSOCIATION_DB_DISEASE	celiac disease; Wegener's granulomatosis; cervical cancer	RT		2	0.4	8.3E-2	8.0E-1
	GENETIC_ASSOCIATION_DB_DISEASE	multiple system atrophy	RT		2	0.4	8.3E-2	8.0E-1
	GENETIC_ASSOCIATION_DB_DISEASE	vitiligo	RT		2	0.4	8.3E-2	8.0E-1
	GENETIC_ASSOCIATION_DB_DISEASE	spondyloarthropathies	RT		2	0.4	9.2E-2	8.1E-1
	GENETIC_ASSOCIATION_DB_DISEASE	copd	RT		2	0.4	9.2E-2	8.1E-1
	GENETIC_ASSOCIATION_DB_DISEASE	dermatitis, atopic	RT		2	0.4	9.2E-2	8.1E-1
	GENETIC_ASSOCIATION_DB_DISEASE	endometriosis	RT		3	0.6	9.3E-2	8.0E-1
	GENETIC_ASSOCIATION_DB_DISEASE	arthritis	RT		3	0.6	9.6E-2	8.0E-1
	GENETIC_ASSOCIATION_DB_DISEASE	stroke, ischemic	RT		3	0.6	9.9E-2	7.9E-1

Muitiple sclerosis genes

OFFICIAL_GENE_SYMBOL	GENE NAME	Related Genes	Species
CTSS	cathepsin S	RG	Homo sapiens
ICAM1	intercellular adhesion molecule 1	RG	Homo sapiens
HLA-B, HLA-C	major histocompatibility complex, class I, C; major histocompatibility complex, class I, B	RG	Homo sapiens
MX1	myxovirus (influenza virus) resistance 1, interferon-inducible protein p78 (mouse)	RG	Homo sapiens
PLP1	proteolipid protein 1	RG	Homo sapiens
HLA-DQA1	similar to hCG2042724; similar to HLA class II histocompatibility antigen, DQ(1) alpha chain precursor (DC-4 alpha chain); major histocompatibility complex, class II, DQ alpha 1	RG	Homo sapiens

Supplementary Figure S1: The disease classes of 66 significant differentially expressed genes were analyzed by DAVID functional annotation chart tool.

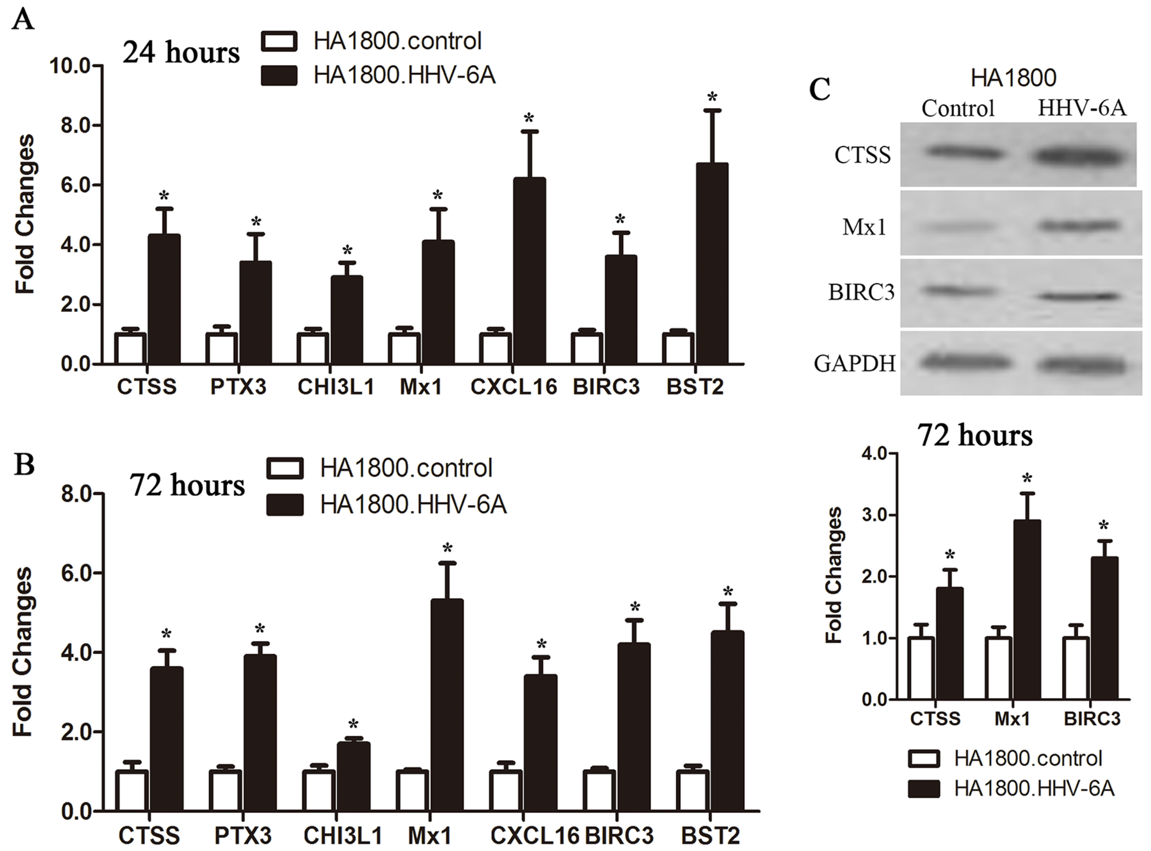

Supplementary Figure S2: The verification of differentially expressed genes in HA1800-HHV6A GS cells. A. Real-time PCR assay for upregulated genes at 24 hours in HA1800-HHV6A GS cells compared to control cells. All data represent mean ± s.e.m. *P < 0.05. (n=4) **B.** Real-time PCR assay for upregulated genes at 72 hours in HA1800-HHV6A GS cells compared to control cells. All data represent mean ± s.e.m. *P < 0.05. (n=4) **C.** Western blot assay for CTSS, Mx1, and BIRC3 genes at 72 hours in HA1800-HHV6A GS cells compared to control cells. All data represent mean ± s.e.m. *P < 0.05. (n=3).

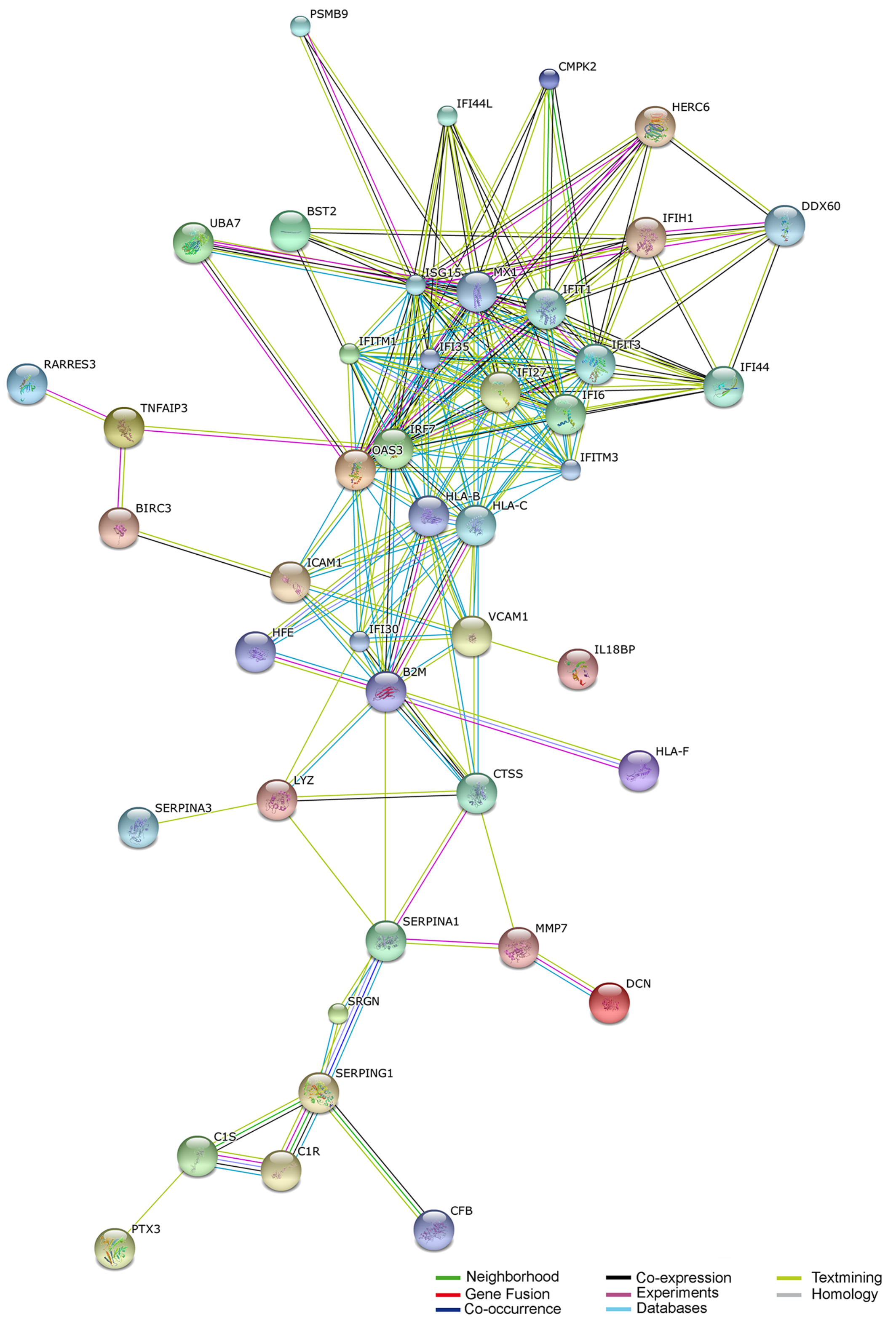

Supplementary Figure S3: The differentially expressed genes (249 genes of FDR < 0.1) exhibiting a positive correlation with viral infection by STRING (http://string.embl.de/).

Supplementary Table S1: List of differentially expressed genes (16430 genes, and 249 genes of FDR < 0.1) between libraries.

See Supplementary File 1

Supplementary Table S2: List of 66 significant differentially expressed genes with FDR < 0.1 (HA1800-control expression > -1 and HA1800-HHV6-GS expression > 0) between libraries.

See Supplementary File 2

Supplementary Table S3: List of differentially expressed genes enriched GO terms for molecular functions.

See Supplementary File 3

Supplementary Table S4: List of differentially expressed genes enriched GO terms for cellular components.

See Supplementary File 4

Supplementary Table S5: List of differentially expressed genes enriched GO terms for biological processes.

See Supplementary File 5

Supplementary Table S6: List of differentially expressed genes enriched GO terms for pathway analysis.

See Supplementary File 6

Supplementary Table S7: List of the primers for real-time PCR

Gene symbols	Primers(5'-3')
**CTSS**	F: TGACAACGGCTTTCCAGTACA<br>R: GGCAGCACGATATTTTGAGTCAT
**PTX3**	F: CATCTCCTTGCGATTCTGTTTTG<br>R: CCATTCCGAGTGCTCCTGA
**CHI3L1**	F: GTGAAGGCGTCTCAAACAGG<br>R: GAAGCGGTCAAGGGCATCT
**Mx1**	F: GTTTCCGAAGTGGACATCGCA<br>R: CTGCACAGGTTGTTCTCAGC
**CXCL16**	F: CCCGCCATCGGTTCAGTTC<br>R: CCCCGAGTAAGCATGTCCAC
**BIRC3**	F: TTTCCGTGGCTCTTATTCAAACT<br>R: GCACAGTGGTAGGAACTTCTCAT
**BST2**	F: CACACTGTGATGGCCCTAATG<br>R: GTCCGCGATTCTCACGCTT
**GAPDH**	F: ACAACTTTGGTATCGTGGAAGG<br>R: GCCATCACGCCACAGTTTC
